# Case of Lumbar Spinal Caries, Psoas Abscess, Suppurative Cerebro-Spinal Meningitis, Suicidal Tendencies. Death, Necropsy

**Published:** 1885-09

**Authors:** Harding H. Tomkins

**Affiliations:** Gloucester County Lunatic Asylum


### CASE OF LUMBAR SPINAL CARIES, PSOAS ABSCESS, SUPPURATIVE CEREBRO-SPINAL MENINGITIS, SUICIDAL TENDENCIES. DEATH, NECROPSY. By HARDING H. TOMKINS, M.R.C.S., Gloucester County Lunatic Asylum.

E. C. J. was admitted to the Gloucester County Asylum on August 28th, 1880. At that time he was passing through his first attack of acute mania, having been ill three months. He was *not* suicidal, but was dangerous to others. His family history, physical and psychological, was good, there being no history of phthisis, of neuroses, or insanity. His own bodily condition was good, and he was free from any physical ailment: he was *not* ruptured on admission. Mentally, he was sullen, morose, irrational, and reticent.

From this attack he recovered partially, only to pass through a recurrent attack in June, 1881, which left him considerably demented.

In February, 1882, he complained of cold and pain in his back, which, however, appeared to pass away under simple treatment.

During March, one day it was discovered that he was ruptured, and subsequently there was slight inflammation in the integuments, necessitating his removal to the sick-ward. The hernia, which was a left inguinal hernia of small size, was easily reduced.

By January, 1883, he had improved bodily, being in fair health and able to work about in the house; he was, however, more demented.

In March (and from that time forward) it was noticed that he acquired a slouching, awkward gait, usually having his hands in his pockets. This, however, was not taken much notice of, in an asylum where so many patients have like peculiarities of gait and appearance. He remained apparently unchanged, bodily or mentally, until,

January 7th, 1885, when the hernia suddenly became about three times its usual size, causing him a certain amount of pain. He was put to bed and kept there, and on the second day strong lead and opium lotion (liq. plumbi. subacet., ℥iv.; tr. opii., ʒii; aq., oj) was applied on account of hyperæmia and increased local temperature. In the course of a few days there was much induration, extending into Scarpa's triangle, with a localized area, soft and fluctuating, evidently containing pus. This was aspirated, about 10 oz. of pus being withdrawn. The skin around the puncture commenced to slough, and the abscess sac was therefore cut into, and the wound dressed with iodoform and carbolic tow. The amount of pus became very small, and the hernia subsided, there never having been signs of obstruction. The drainage tube was removed; then, however, pus collected in the iliac fossa of the left side, which was let out, under chloroform, by freely incising the original wound, a drainage tube also being passed under Poupart's ligament, and another deep amongst the muscles of the thigh, which were lying dissected out by the pus. The little finger passed with ease through the inguinal canal into the abdomen. About a pint of pus escaped, the patient being placed in the prone position.

For a few days the amount of pus diminished, and there was none discovered in the abdomen. The cavity was syringed daily with solution of boro-glyceride (5 %), and dressed as before, antiseptic surgery being impossible, as the man frequently removed all dressings. One night, when making my night round, he begged me to take care of a purse he had, containing a shilling or two, as he said he should never want it again. Being struck by the earnestness of the request, and the man's appearance, I felt sure he contemplated suicide; therefore I sent one of the night attendants to remain with him, who discovered that he had suddenly become intensely suicidal. On visiting him soon after, I found him extremely agitated—his lips compressed, nostrils dilated, and whole expression very wild, his pulse also beating at 140. He begged and implored me to kill him, saying that if I did not, he must do it himself. When, after trying for a long time to pacify him, I told him I must not kill him, he suddenly sprang up in bed, cursing and swearing, and vowing he *would* kill himself, and that he would overpower us if we tried to prevent him. It took three men to put him in a padded room, where, after $\frac{1}{2}$ grain of morphia hypodermically, one attendant remained with him all night, he getting scarcely any sleep. From this time till death he remained determinately suicidal. He now complained of pain in his knee and the front of his thigh on the left side; pus, also, accumulated in the abdomen, pointing in Scarpa's triangle and to the outer side of the tuber ischii on the *right side*, in which latter position an opening was made, under chloroform, on March 7th, 1885, 2½ pints of pus being evacuated.

After the second operation he fell away rapidly, the amount of pus being very large daily. He also complained of pain in his lumbar region for the first time since February, 1882; there was, however, no tenderness or apparent deformity, though on several occasions he was examined for spinal curvature. He died on March 20th, apparently from exhaustion. His appetite remained good up to the last. His temperature could not be ascertained at any time by the thermometer; but it appeared never to rise to any considerable extent.

*Post-mortem Examination.*—Brain, 44½ oz.; substance anæmic and somewhat atrophied. The membranes presented the usual appearance of acute meningitis, the base of the brain being also covered with pus, and *all the ventricles* contained pus.

Spinal cord was everywhere surrounded by pus, the membranes being hyperæmic and much thickened.

Spinal vertebræ: The second and third lumbar vertebræ were in an advanced state of caries centrally; but the external surfaces appeared healthy; hence no deformity was produced. The inter-vertebral disc had been absorbed. There were two large abscesses in both psoas regions, communicating with the spinal canal between the second and third lumbar vertebræ.

Right lung was consolidated at the apex, and presented many tubercles.

It is interesting that so advanced a state of caries and abscess could exist (commencing probably February, 1882), the patient yet retaining power and ability, not only to work for so long without evidence of pain, &c., but also to fight so actively when trying to get an opportunity to kill himself. The diagnosis was made particularly hard because the only symptom which might have aroused suspicion before the formation of pus (viz., his peculiar gait) was of so little account amongst lunatics, who assume all kinds of positions and carriage without any apparent reason; and also because when an abscess did form, it appeared to be accounted for at first by the sudden tearing up of the cellular tissue by the enlargement of a hernia, which was followed by induration and suppuration. It is probable that the hernia was caused directly by the pressure of the pus from within, and, from the history of the case, that the two abscesses were originally distinct, but that one having formed externally and destroyed the superficial structures, the psoas abscess broke into the cavity already formed, a channel having been opened by the hernia subsiding. A more interesting point is the evidence of so much mischief in the cerebro-spinal system, with but few symptoms to indicate it, and these such as can only be recognised by looking back after the close of the case. The pupils exhibited no change whatever; the only sign of change of power or sensation was slight stiffness and pain in the lower jaw. There was no mental symptom except the sudden change to violent suicidal tendency from sullenness and dementia; and even this is not unusual in recurrent maniacs, the symptoms changing in different attacks. There were no convulsions; nor was there coma, the patient apparently dying of simple exhaustion.

It seems probable that the paucity of symptoms may in part be explained by the previous attacks, extending over five years, having resulted in dementia, accompanied by wasting of the brain, so that the abnormal amount of fluid produced no compression of the brain tissue, which in its turn was altered so as to render it less sensitive to such changes as those discovered at the *post-mortem*. Also the amount of pus present was not necessarily a measure of the extent or acuteness of the inflammation, since doubtless a good deal was merely the result of gravitation from the spinal abscess, although the membranes presented appearances sufficient to warrant a diagnosis of meningitis.

